# HbWRKY27, a group IIe WRKY transcription factor, positively regulates *HbFPS1* expression in *Hevea brasiliensis*

**DOI:** 10.1038/s41598-020-77805-5

**Published:** 2020-11-26

**Authors:** Long Qu, Hui-Liang Li, Dong Guo, Ying Wang, Jia-Hong Zhu, Li-Yan Yin, Shi-Qing Peng

**Affiliations:** 1 School of Life and Pharmaceutical Sciences, Hainan University, Haikou, 570228 China; 2 Key Laboratory of Biology and Genetic Resources of Tropical Crops, Ministry of Agriculture, Institute of Tropical Bioscience and Biotechnology, Chinese Academy of Tropical Agricultural Sciences, No.4 Xueyuan Road, Haikou, 571101 China

**Keywords:** Molecular biology, Plant sciences

## Abstract

Farnesyl pyrophosphate synthase (FPS) is a key enzyme that catalyzes the formation of farnesyl pyrophosphate, the main initiator for rubber chain initiation *in Hevea brasiliensis* Muell. Arg. The transcriptional regulatory mechanisms of the FPS gene still not well understood. Here, a WRKY transcription factor designated HbWRKY27 was obtained by screening the latex cDNA library applied the *HbFPS1* promoter as bait. HbWRKY27 interacted with the *HbFPS1* promoter was further identified by individual Y1H and EMSA assays. HbWRKY27 belongs to group IIe WRKY subfamily which contains a typical WRKY domain and C-X5-CX23-HXH motif. HbWRKY27 was localized to the nucleus. *HbWRKY27* predominantly accumulated in latex. *HbWRKY27* was up-regulated in latex by ethrel, salicylic acid, abscisic acid, and methyl jasmonate treatment. Transient expression of HbWRKY27 led to increasing the activity of the *HbFPS1* promoter in tobacco plant, suggesting that HbWRKY27 positively regulates the *HbFPS1* expression. Taken together, an upstream transcription factor of the key natural rubber biosynthesis gene *HbFPS1* was identified and this study will provide novel transcriptional regulatory mechanisms of the FPS gene in *Hevea brasiliensis*.

## Introduction

Rubber tree (*Hevea brasiliensis* Muell. Arg.) is an important rubber-producing plant of *Euphorbiaceae*^1,2^*.* The valuable of rubber tree as a sole commercial source rubber-producing plant led to enormous interest in understanding the natural rubber biosynthesis and regulation in rubber tree. Natural rubber is produced from the latex which is a complex cytoplasmic system of laticifers in the rubber tree^3^. Generally, natural rubber *cis* 1, 4-polyisoprene biopolymer, is mainly synthesized by the mevalonate pathway that produces isopentenyl pyrophosphate (IPP) as the precursor and building rubber chain skeleton^1^. The biosynthetic pathway of natural rubber can be divided into three stages: initiation, polymerization, and termination^4^. Farnesyl pyrophosphate (FPP) may be the main initiator during natural rubber biosynthesis in rubber tree^5–7^. The concentration of FPP and the ratio of FPP and IPP affect the rubber biosynthetic rate and rubber molecular weight^8^. Farnesyl pyrophosphate synthase (FPS) catalyzes the consecutive head-to-tail condensations of geranyl pyrophosphate or dimethylallyl diphosphate with two molecules of IPP to form FPP^9,10^. Thus, FPS should be considered as a crucial enzyme in the natural rubber biosynthesis. The rubber tree FPS genes (named *HbFPS1*, *HbFPS2*, and *HbFPS3*) have been cloned and characterized^11^. The expression of *HbFPS1* exhibits a positive correlation with natural rubber biosynthesis^11,12^. Recently two MYB transcription factors (HblMYB19 and HblMYB44) are identified to up-regulate the expression of *HbFPS1*^13^. However, the regulatory mechanisms of the *HbFPS1* expression still remain poorly understood. Here, a WRKY transcription factor (designated as HbWRKY27) bound the *HbFPS1* promoter and positively regulate *HbFPS1* expression, demonstrating that HbWRKY27 might a positive transcription regulator of *HbFPS1*.

## Materials and methods

### Plant materials

Rubber tree cultivar CATAS 7-33-97, planted in the experimental plantation of the Chinese Academy of Tropical Agricultural Sciences, was employed to harvest different samples including latex, leaves, flowers, roots, and bark as described previously^11^. Rubber tree shoots were treated by 0.5% abscisic acid (ABA), 0.2% salicylic acid (SA), 0.07% methyl jasmonate (JA), and 0.5% Ethrel (ET) in accordance with Hao and Wu’ method^14^. Five groups (10 trees in each group) were employed in each treatment, in which the plant hormone was applied at 3, 6, 9, 12, and 24 h before tapping. The other group was not treated with hormone as control. After the treatments at all time points, latex from all the tested trees were collected. Latex from the same group was mixed together thoroughly. The resulting solution was then divided into five equal volumes for RNA extraction. *N*. *benthamiana *seeds were sowed on moist filter paper in a glass garden, and then incubate them in a growth chamber maintained at a relative humidity of 60–70%, a temperature of 28 °C, and 14 h day/10 h night cycle. After a week, the seedlings were potted in soil and placed in a greenhouse maintained at 26–28 °C, a relative humidity of 60–70%, and 14 h day/10 h night cycle. Two months old seedlings were used to test.

### Isolation of DNA and RNA

Genomic DNA isolated from young leaves of CATAS 7-33-97 using the Plant Genomic DNA Extraction Kit (TaKaRa, Dalin, China). Isolation of total RNA from different samples was carried out in accordance with the method of Wang et al.^15^.

### Yeast one-hybrid (Y1H) assay

The *HbFPS1* promoter fragment (1066 bp) was cloned by PCR with primers (Table 1) using the Genomic DNA as the template in accordance with described method^10^. Then the *HbFPS1* promoter fragment was inserted in bait vector pHIS2.1, generate the pHIS-pHbFPS1 construct. Latex cDNA library was constructed in accordance with the user manual of Matchmaker Gold Yeast One-Hybrid Library Screening System Kit (Clontech, CA, USA). The screening was performed according to the protocol of Matchmaker Gold Y1H System (Clontech, CA, USA). More than 1 × 10^6^ clones were screened and the positive clones were sequenced and analyzed. 35 transformants were obtained and 22 positive colonies were further obtained after re-streaking the primary positive colonies on the same selective medium. These colonies were further analyzed by plasmid rescue followed by sequence analysis.

**Table 1:** List of primers used in this study.

Usage	Vector	Forward (5′–3′)	Reverse (5′–3′)	Restriction site
Promoter amplified		ATTCAAAATACAAGTTGATTAGG	GGATTCAAACGGAGATTAGATG	
Promoter mutated (internal mutagenic primer)		GCCTTGAGAGTTGAAACCTCTGCAT		
Y1H	pHIS-HbFPS1	CGGAATTCATTCAAAATACAAGTTGATTAGG	CGGAGCTCGGATTCAAACGGAGATTAGATG	*Eco*R I, *Sac* I
	pGAD-HbWRKY27	ACACATATGCTCTCCATGGCTGAGGACTG	TGTGGATCCGTCACCCCCTACGACGGCTGAG	*Nde* I, *Bam*H I
qPCR		TGATATTCTGATTCCTAACATGA	AACCGGCTTAGTGAGAAATTT	
Subcellular localization	pET-HbWRKY27	GTAGATCTGATGCTCTCCATGGCTGAGGACTG	TTTACTAGTGTCACCCCCTACGACGGCTGAG	*Bgl* II, *Spe* I
Reporter vector	pGreen-HbFPS1(M)	GCGCTCGAGTACGAATTCATACAAGTTGAT	GCGGGATCCCAGGTCGACCAAAC GGAGATT	*Xho* I, *Bam*H I
Effector vector	p1301-HbWRKY27	ACACCATGGATGCTCTCCATGGCTGAGGACTG	TGTCACGTGGTCACCCCCTACGACGGCTGAG	*Nco* I, *Pml* I

The interaction between HbWRY27 and the promoter of *HbFPS1* was further confirmed by individual Y1H assays. *HbWRKY27* was amplified by PCR (see Table 1 for PCR primers) and cloned into the prey vector pGADT7-Rec2 to generate pGADT7-HbWRKY27. pHIS-pHbFPS1 and pGADT7-HbWRKY27 were co-transformed into the yeast Y187 strain. p53-HIS2, pGAD-Rec2-53, as well as pHIS-pHbFPS1 were employed as control. The introduced cells was examined on SD/-Leu-His plates and triple dropout (TDO) plates (SD/-Trp-His-Leu ) supplemented with 80 mM 3-amino-1,2,4-triazole (3-AT) for 5 d at 28 °C.

### Electrophoretic mobility shift assay (EMSA)

The full length cDNA of *HbWRY27* was cloned by PCR (primers see Table 1). The cDNA fragment was cloned into the vector pET-28a, and then introduced into *Escherichia coli* strain BL21 to product the HbWRKY27 recombinant proteins according to the user manual (Novagen, Madison, WI, USA). The DNA–protein binding reaction was performed by incubating double-stranded DNA of the *HbFPS1* promoter or the mutated promoter with purified recombinant protein at room temperature. The W-box in the *HbFPS1* promoter was mutated (changing TTGAC to TTGAA) by single-tube ‘megaprimer’ PCR method^16^. EMSA was performed with SYBR Green and SYPRO Ruby EMSA stains as described manufacturer’s protocol of EMSA kits (Invitrogen, Carisbad, CA, USA).

### Phylogenetic analysis

The homologous protein sequences of HbWRKY27 were obtained from GenBank and phylogenetic analysis was carried out based on the neighbor-joining method using MEGA 5.0 software^16^.

### Subcellular localization

The cDNAs of *HbWRKY27* was amplified by PCR with the primers (Table 1) and inserted into the pCAMBIA1302 vector containing the green fluorescent protein (GFP) gene, thereby generating pHbWRKY27-GFP. pHbWRKY27-GFP and pCAMBIA1302 vector were transformed into *A. tumefaciens* strain GV3101 via electroporation. Then *A. tumefaciens* harboring pCAMBIA1302 or pHbWRKY27-GFP were transformed into onion epidermis by infiltration as previously described^17^. Introduced onion epidermal cells was analyzed at 2 days after cultured on MS medium. Fluorescence and 4′, 6′-diamidino-2-phenylindole hydrochloride (DAPI) staining were monitored under a confocal microscope (Leica, Wetzlar, Germany).

### Expression analyses of *HbWRKY27*

Expression of *HbWRKY27* was analyzed by real-time qPCRs in accordance with the manufacturer’s instruction of SYBR Premix Taq Kit (TaKaRa, Dalin, China). *HbACTIN7* was used as a control gene as described previously^17^. The relative expression level of *HbWRKY27* was calculated using the 2^−ΔΔCT^ method^19^. Three biological repeats were carried out. Data are presented as mean ± SE (n = 3).

### Transient expression assay

The *HbFPS1* promoter was cloned by PCR (primers see Table 1) and the *HbFPS1* promoter and the mutated promoter fragment was inserted into the pGreenII 0800-LUC vector, generating a reporter construct pGreenII-pHbFPS1-LUC and pGreenII-pHbFPS1M-LUC. To generate effector gene, *HbWRKY27* was also cloned through PCR primers (Table 1) and inserted into pCAMBIA1301. The generated *HbFPS1-LUC HbFPS1M-LUC* construct and the effector construct, was introduced into tobacco leaves as previously described^20^. The dual-luciferase (LUC) assay was performed according to the manufacturer’s protocol of a dual-luciferase reporter assay system (Promega, Fitchburg, WI, USA). More than three biological repeats were carried out. Difference was accepted as significant at P ≤ 0.05.

## Results

### HbWRY27 interacts with *HbFPS1* promoter

**Figure 1:**
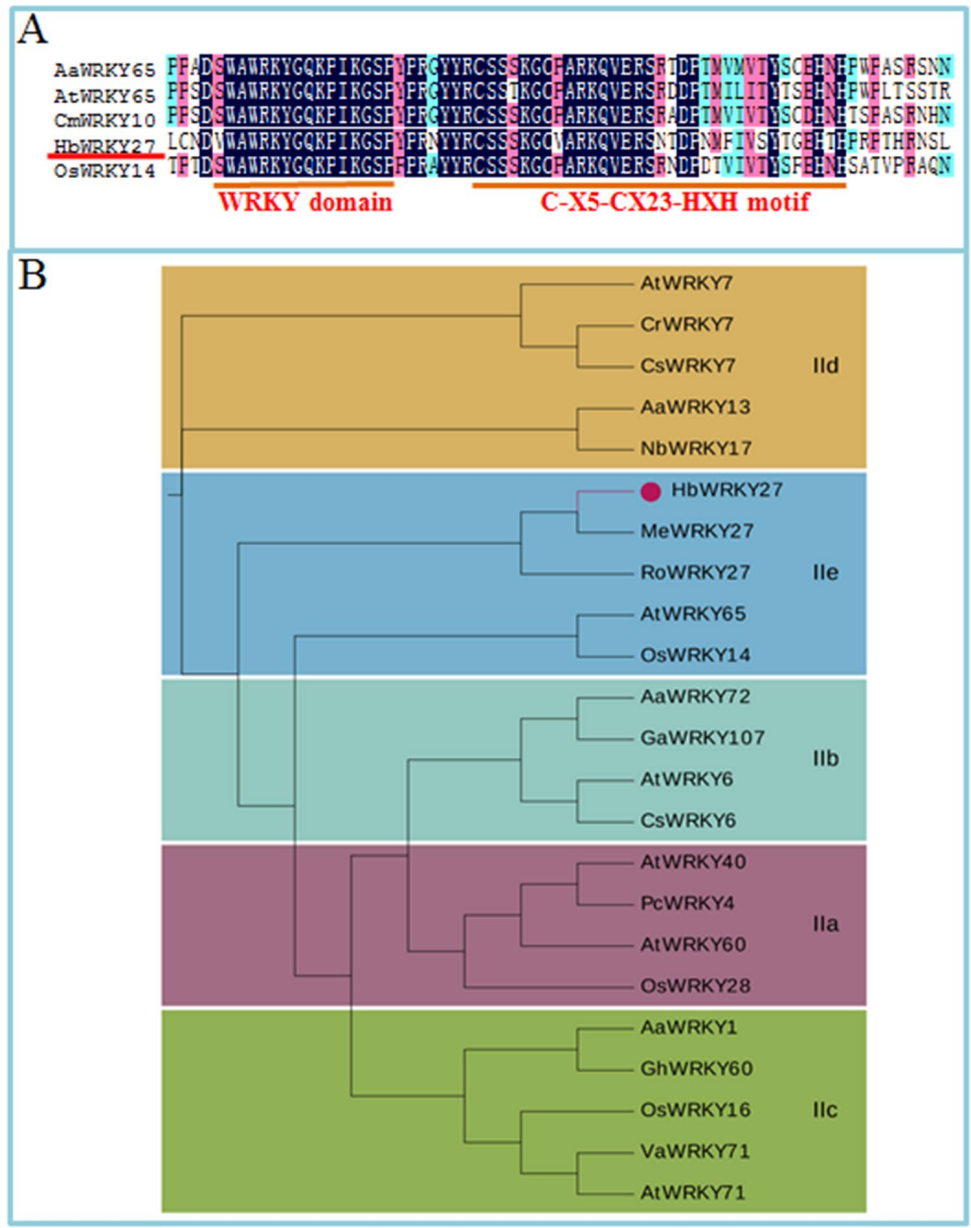
Alignment of the deduced HbWRKY27 protein sequences. (**A**) WRKY domain (WRKYGQK) and C-X5-CX23-HXH motif of the HbWRKY27. (**B**) A phylogenetic tree of the HbWRKY27 proteins and other plants group IIe WRKYs was constructed based on the neighbor joining method, including AaWRKY1 (PWA39112), AaWRKY13 (PWA69470), AaWRKY65 (PWA83388), AaWRKY72 (PWA39515), AtWRKY6 (Q9C519), AtWRKY7 (ANM67919), AtWRKY28 (AEE84006), AtWRKY40 (AEE36457), AtWRKY60 (ANM63193), AtWRKY65 (AEE31068), AtWRKY71 (AEE31143), AtWRKY74 (AED93824), CmWRKY10 (AHC54615), CsWRKY6 (AYA73384), GaWRKY107 (AIY62483), GhWRKY60 (AGV75958), NbWRKY17 (AIR74899), OsWRKY14 (DAA05079), OsWRKY16 (DAA05081), OsWRKY28 (Q0DAJ3), OsWRKY32 (DAA05097), OsWRKY49 (DAA05114), OsWRKY68 (DAA05133), PcWRKY4 (AAG35658), VaWRKY71 (AFK27602).

**Figure 2:**
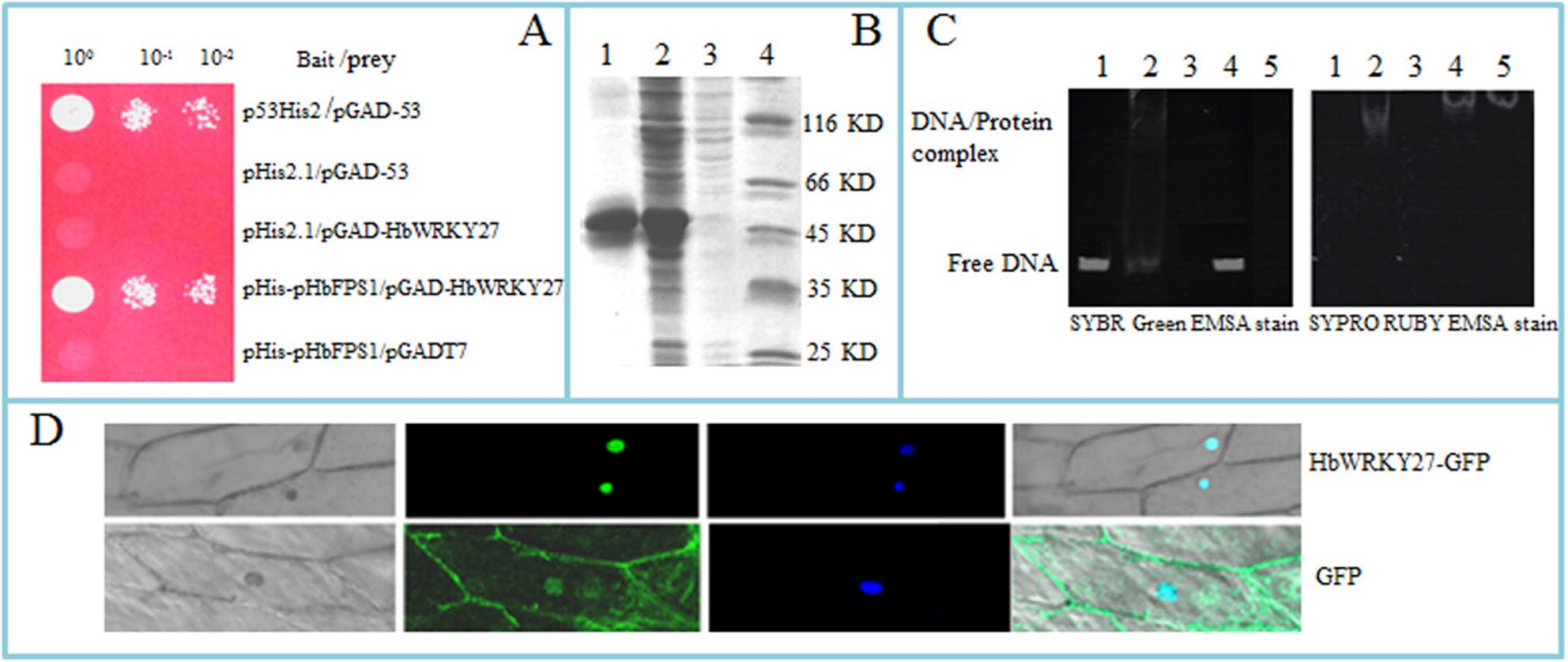
Characterization of the HbWRKY27. (**A**) Y1H assays of the binding specificity of the *HbFPS1* promoter with HbWRKY27. The yeast cells were cultured on a medium lacking leucine, tryptophan, and histidine (SD/–Trp/–Leu/–His) supplemented with 80 mM 3-amino-1,2,4-triazole (3-AT). Panels show yeast serial decimal dilutions. (**B**) Heterologous expressing of *HbWRKY27* in *E. coli*. 1. Purified HbWRKY27 fusion protein, 2. *E. coli* cells harboring pET-HbWRKY27 after 3 h of induction, 3. *E. coli* cells harboring pET-HbWRKY27 not induced, 4. Molecular markers. (**C**) Analysis of the binding ability of the *HbFPS1* promoter with HbWRKY27 was analyzed via electrophoretic mobility shift assay (EMSA). In the left panel, the gel was stained to visualize the DNA with a SYBR green stain. In the right panel, the gel was stained to monitor the proteins with a SYPRO Ruby EMSA stain. Lane 1. The promoter of *HbFPS1* DNA (300 ng) only. Lane 2. HbWRKY27 protein (400 ng) with the promoter of *HbFPS1* DNA (300 ng). Lane 3. The mutated promoter of *HbFPS1* DNA (300 ng) only. Lane 4. HbWRKY27 protein (400 ng) with the mutated promoter of *HbFPS1* DNA (300 ng). Lane 5. HbWRKY27 protein (400 ng) only. (**D**) Subcellular localization of HBWRKY27-GFP fusion protein in onion epidermal cells. GFP was used as a control and DAPI staining as a nuclear marker.

The *HbFPS1* promoter had been cloned in previous study^21^. To understand the transcriptional regulatory of *HbFPS1*, the *HbFPS1* promoter was employed as the bait to screen transcription factors that interact with the *HbFPS1* promoter from Y1H-based latex cDNA library. Twenty-two colonies were obtained and sequenced. Eight candidates encoding transcription factors were obtained (Supplementary Information Table S1). Among of candidates, one cDNA encoding WRKY transcription factor, named *HbWRKY27* according to its homologs in Genbank, was obtained. *HbWRKY27* had an open read frame of 1266 bp in length. The molecular mass of the deduced HbWRKY27 protein was 46.9 kDa. HbWRKY27 had a typical WRKY domain and C-X5-CX23-HXH motif (Fig. 1A). HbWRKY27 was classified into group IIe WRKY subfamily (Fig. 1B). A binding site (W-box) for WRKYs in the promoter of *HbFPS1* was predicted^21^. HbWRKY27 interacted with *HbFPS1* promoter was further identified by individual Y1H assays (Fig. 2A). To further determine that HbWRKY27 physically bound with the *HbFPS1* promoter, EMSA was used to confirm the binding affinity of HbWRKY27 to the *HbFPS1* promoter. The recombinant HbWRKY27 protein was obtained by heterologous expressing of *HbWRKY27* in *E. coli* (Fig. 2B). In addition, the W-box in the *HbFPS1* promoter was mutated (changing TTGAC to TTGAA) by PCR method. The DNA–protein binding signal was detected with the recombinant HbWRKY27 protein incubated with the *HbFPS1* promoter. No binding signal was detected with the mutated *HbFPS1* promoter (Fig. 2C). The result of EMSA also displayed HbWRKY27 interacted with *HbFPS1* promoter and the TTGAC is necessary for binding of HbWRKY27 protein to the *HbFPS1* promoter.

### Subcellular localization of HbWRKY27

To elucidate the subcellular localization of HbWRKY27, the green fluorescent protein (GFP) gene was employed as a marker to fuse *HbWRKY27* in-frame, generating the HbWRKY27-GFP construct. Compared with the fluorescence was clearly visible in the cytoplasm and nucleus of the cell transformed with 35S-GFP, the fluorescence was restricted to the nucleus of the cell transformed with HbWRKY27-GFP (Fig. 2D), suggesting that HbWRKY27 was a nuclear-localized protein.

### Expression profile of *HbWRKY27*

**Figure 3:**
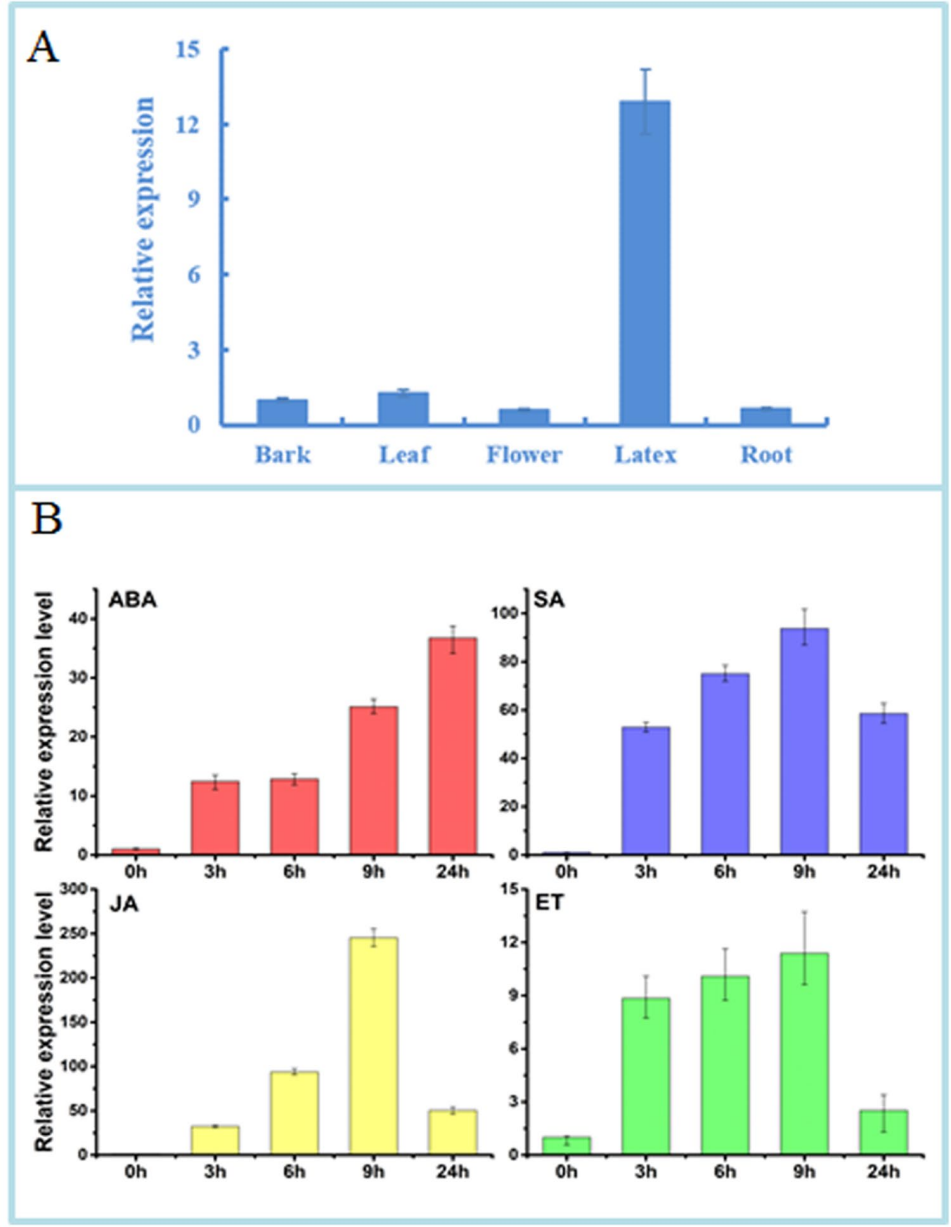
Transcription profiles of *HbWRKY27*. (**A**) Expression patterns of *HbWRKY27* in rubber tree. Transcript abundances in different tissues are expressed relative to the level in bark. Data are presented as mean ± SE (n = 3). (**B**) Expression patterns of *HbWRKY27* responding to ABA, SA, ET and JA treatment in latex. Transcript abundances in different tissues are expressed relative to the level in control. Data are presented as mean ± SE (n = 3).

Expression of *HbWRKY27* was analyzed by qPCR. The result of qPCR showed that *HbWRKY27* predominantly accumulated in latex, but little expression was detected in the leaves, flowers, roots, and barks (Fig. 3A). To investigate *HbWRKY27* expression in response to ABA, SA, ET, and JA treatment in latex, qPCR analysis of *HbWRKY27* expression were carried out. The expression of *HbWRKY27* was up-regulated by ABA, SA, ET, and JA treatment. The expression of *HbWRKY27* reached its maximum level after 9 h of SA, ET, and JA treatment, while the expression of *HbWRKY27* reached its maximum level after 24 h of ABA treatment (Fig. 3B).

### HbWRKY27 activates the transcription of *HbFPS1*

**Figure 4:**
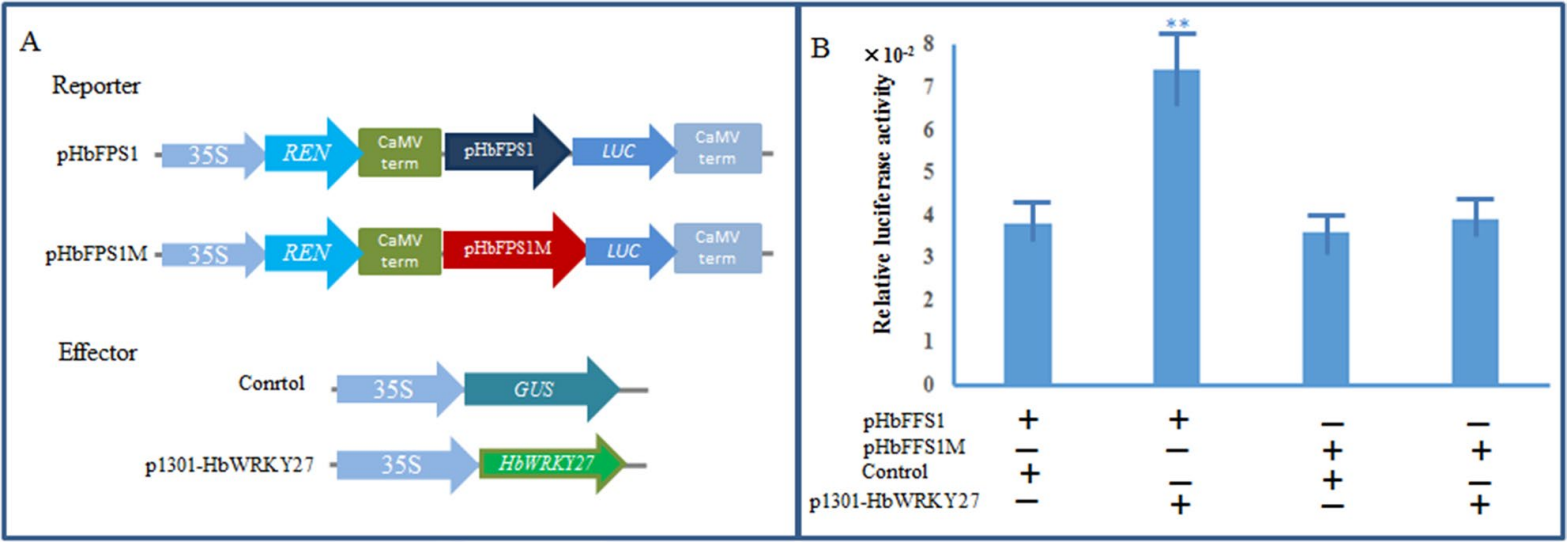
Activation of *HbFPS1* promoter by HbWRKY27. (**A**) Schematic drawing of the reporter and effector construct. (**B**) Effect of HbWRKY27 on the activation of the *HbFPS1* promoter. The relative LUC activities (LUC/REN) were normalized to the reference *Renilla* (REN) luciferase. Error bars indicate SE from five biological replicates (**p < 0.01).

To further study the regulatory relationship of HbWRKY27 and the transcription of *HbFPS1*, the *luciferase* (*LUC*) was employed as a report gene to fuse with the *HbFPS1* promoter fragment to generate the pHbFPS1:LUC construct, and the effector p35S-HbWRKY27 was constructed (Fig. 4A). pHbFPS1:LUC was introduced into tobacco leaves along with p35S-GUS or p35S-HbWRKY27. Dual-luciferase assays indicated that HbWRKY27 had significant activation effect on transcription from the *HbFPS1* promoter and had no activation effect on transcription from the mutated *HbFPS1* promoter (Fig. 4B), indicating that HbWRKY27 could bind the *HbFPS1* promoter and activate the *HbFPS1* promoter in the transcription level.

## Discussion

*FPSs* have been identified in a few plants^11,22,23,25^. *FPSs* belong to a small multigenic family which encodes at least two different isoforms in plants. The members of the *FPS* family have a different pattern of expression that vary among different plant species^11,22^. For example, in Arabidopsis *FPS1* is predominantly expressed in roots and inflorescences, whereas *FPS2* accumulates preferentially mRNA in inflorescences^22^. In *Ginkgo biloba*, the higher *GbFPS* expression level was detected in roots and leaves^23^, in which the ginkgolides and bilobalide are synthesized^24^. In *Euphorbia pekinensis*, *EpFPS* had a high transcription level in roots, in which terpenoids are synthesized^25^. In the rubber tree, *HbFPS1* is expressed predominantly in the laticifers and is likely to encode the enzyme involved in natural rubber biosynthesis^11^. To our knowledge, the transcriptional regulatory mechanisms of FPS gene in plant has not been reported.

WRKY transcription factors, a plant specific transcription factor family, play crucial roles in plant secondary metabolites^26–28^. For example, GaWRKY1 regulates the biosynthesis of gossypol in *Gossypium* spp^29^. AaWRKY1 regulates the biosynthesis of artemisinin in *Artemisia annua *^30^. In *Vitis vinifera*, VviWRKY40 modulates glycosylated monoterpenoid production^31^. The phylogenetic analyses revealed that HbWRKY27 is highly homologous with MeWRKY27, RoWRKY27, AtWRKY65, and OsWRKY14. OsWRKY14 regulates serotonin production through the up-regulation of the expression of tryptophan synthase gene and tryptophan decarboxylase gene in rice^32^. The role of other homologs of HbWRKY27 has never been reported. More than 80 WRKY proteins in rubber tree have been identified^33^. HbWRKY1 is demonstrated to repress the expression of *HbSRPP,* a natural rubber biosynthesis-related gene, suggesting HbWRKY1 might a negative regulator in natural rubber biosynthesis^15^. Over-expressing of HbWRKY40 in Arabidopsis increased resistance against *Botrytis cinerea*^34^. Except these, the function of few HbWRKYs had been reported. Here, Y1H and EMSA analysis displayed HbWRKY27 bound the *HbFPS1* promoter. *HbFPS1* is predominantly expressed in latex where natural rubber is synthesized^11,12^. Intriguingly, *HbWRKY27* was also predominantly accumulated in latex, consisting with the expression profile of *HbFPS1*, suggesting the co-ordinate regulation of natural rubber biosynthesis by both *HbWRKY27* and *HbFPS1.* Moreover transient expression of HbWRKY27 led to increase the activity of the *HbFPS1* promoter in vivo, suggesting HbWRKY27 might a positive regulator in natural rubber biosynthesis.

In rubber tree, the natural rubber biosynthesis pathway underlying enzymes have been identified^35,36^, but the transcriptional regulatory of rubber biosynthesis are poorly understood^37–39^. A few transcription factors except WRKYs had been identified to regulate natural rubber biosynthesis-related gene. For example, HbMADS4 has been identified to negatively regulate *HbSRPP* expression^17^*,* while HbMYC2b positively regulates *HbSRPP* expression^40^. HbCZF1 up-regulates 3-hydroxy-3-methyl-glutaryl coenzyme A reductase (HMGR) gene expression^41^. HblMYB19 and HblMYB44 have been identified to up-regulate the *HbFPS1* expression^13^. HbRZFP1 down-regulates rubber transferase gene (*HRT2*) expression. The interaction of 14-3-3 protein with HbRZFP1 led to relieve HbRZFP1-mediated HRT2 transcription inhibition^42^. Even so, the underlying transcriptional regulatory mechanisms of natural rubber biosynthesis are largely unknown. Further investigation of regulatory machinery of natural rubber biosynthesis will be important in manipulating natural rubber metabolism.

Plant hormones have crucial important roles in regulating natural rubber biosynthesis^39,43,44^. WRKY transcription factors are involved in SA, AB, ET, and JA signaling pathways and plays a vital role in the signal crosstalk of the SA, AB, ET, and JA signaling pathways^23,45,46^. The promoter of *HbWRKY27* had a few cis-acting elements related to hormone responses and *HbWRKY27* is simultaneously up-regulated by SA, AB, ET, and JA, suggesting that HbWRKY27 might integrate plant hormones signals and regulates natural rubber biosynthesis. Further investigation should be carried out to study the mechanisms by which HbWRKY27 integrates plant hormones signals and mediates natural rubber biosynthesis.

## Conclusion

In the present study, HbWRKY27 was identified to bind the *HbFPS1* promoter. HbWRKY27 had significant activation effect on transcription from the *HbFPS1* promoter. HbWRK27 might a positive regulator of *HbFPS1*, which participates in natural rubber biosynthesis.

## Supplementary information

Supplementary Information.
