# Supplementary material for: HbWRKY27, a group IIe WRKY transcription factor, positively regulates HbFPS1 expression in Hevea brasiliensis
Source: Sci Rep. 2020 Nov 26;10:20639. doi: 10.1038/s41598-020-77805-5 (PMC7692525; doi:10.1038/s41598-020-77805-5)
Supplement: Supplementary file 1 — Supplementary Information. [file 41598_2020_77805_MOESM1_ESM.pdf]

HbWRKY27, a group IIe WRKY transcription factor, positively regulates *HbFPS1* expression in *Hevea brasiliensis*

Long Qu[1, 2]†, Hui-Liang Li[2]†, Dong Guo[2], Ying Wang[2], Jia-Hong Zhu[2], Li-Yan Yin[1*], Shi-Qing Peng[2*]

[1] School of Life and Pharmaceutical Sciences, Hainan University, Haikou, 570228, China

[2]Key Laboratory of Biology and Genetic Resources of Tropical Crops, Ministry of Agriculture, Institute of Tropical Bioscience and Biotechnology, Chinese Academy of Tropical Agricultural Sciences, Haikou 571101, China

† These authors contributed equally to this work.

* Correspondence: Li-Yan Yin, School of Life and Pharmaceutical Sciences, Hainan University, Haikou, 570228, China. E-mail: lyyin@163.com; Shi-Qing Peng, Key Laboratory of Biology and Genetic Resources of Tropical Crops, Ministry of Agriculture, Institute of Tropical Bioscience and Biotechnology, Chinese Academy of Tropical Agricultural Sciences, No.4 Xueyuan Road, Haikou 571101, China. E-mail: shqpeng@163.com

Table S1 The candidates encoding transcription factors

Number	Candidates	Homologous species
RD01	AP2/ERF domain-containing transcription factor (DREB25)	*Populus trichocarpa*
RD03	Transcription factor MYC2-like	*Cucumis sativus*
RD08	C3HC4-type RING zinc finger protein (RGZF1)	*Hevea brasiliensis*
RD12	GATA transcription factor	*Hevea brasiliensis*
RD17	WRKY transcription factor 27	*Hevea brasiliensis*
RD20	Transcription factor bHLH144	*Hevea brasiliensis*
RD21	WRKY transcription factor 61	*Hevea brasiliensis*
RD27	AP2/ERF domain-containing transcription factor	*Hevea brasiliensis*
